# The Impact of Service Firm’s Environmentally Friendly Reputation in the Context of Revenue Management

**DOI:** 10.3390/ijerph17176264

**Published:** 2020-08-28

**Authors:** Choongbeom Choi, Miyoung Jeong

**Affiliations:** 1 College of Hospitality and Tourism, Sejong University, Seoul 05006, Korea; 2 College of Hospitality, Retail and Sport Management, University of South Carolina, Columbia, SC 29425, USA; jeongm@mailbox.sc.edu

**Keywords:** environmentally friendly reputation, revenue management, perceived fairness, rate framing, rate fencing

## Abstract

In revenue management practice, customers’ perceived fairness is a critical issue. Prior research examined the effect of revenue management on customers’ perceptions of fairness by implementing two different conditions: fencing and framing. In this study, the authors evaluated the role of a service firm’s environmentally friendly reputation under the conditions of fencing and framing. Results indicated that an environmentally friendly reputation only moderated the effect of framing on perceived fairness. In particular, when the firm had a poor reputation, framing as a discount rather than framing as a surcharge increased customers’ fairness perceptions. When the firm had a good reputation, however, customers’ perception of fairness did not differ across two framing conditions. The findings of this study help firms to understand how customers perceive fairness in revenue management practice.

## 1. Introduction

Revenue management (RM) is one of the most commonly adopted practices in the hospitality and airline industry as a way to maximize profits by selling perishable assets such as hotel rooms and airline seats, under the mechanism of controlling prices and inventories [1,2]. However, immediate attention must be paid when implementing this practice because it potentially generates issues related to price fairness perceived by customers who have purchased the service that could be charged differently from others [3]. A firm’s pricing variability or discrepancy for the same service can also cause customers’ distrust and dissatisfaction with the way the company practices [4,5,6] and ultimately lead them to scrutinize underpinning reasons for the firm’s pricing strategies [7,8].

Among various pricing strategies, the two most prevalently used techniques are rate fence and framing, which are known to affect customers’ perceived fairness [3,6]. Kim and Wirtz [6] find that properly designed rate fences assist service providers in effectively charging lower prices for those who are willing to accept certain restrictions on their purchase. They also show that customers are more favorable toward a rate framed as a discount than a rate presented as a surcharge when price fairness comes into play [3]. In addition, prior research documents whether there can be a crucial factor that moderates the effects of fencing and framing conditions on customers’ fairness perceptions. For example, Wirz and Kimes [3] show that the effects of fencing and framing on perceived fairness in RM are moderated by customers’ familiarity with RM.

However, little research has been carried out on whether a firm’s environmentally friendly reputation affects perceptions of RM practice [9]. Previous research argues that a firm’s environmentally friendly reputation can be established when a firm takes its environmental responsibility [10]. When a firm has a good environmentally friendly reputation, such reputation earns more customer leniency in response to the firm’s action [11]. Since price discrimination is inherent in RM, therefore, understanding how a firm’s environmentally friendly behavior alter customers’ fairness perceptions is crucial [9]. In an attempt to address this gap, the current study takes a service firm’s environmentally friendly reputation into consideration as a moderator under the conditions of rate fences and framing in RM practice to better understand customers’ perceived price fairness. It is believed that a service provider’s reputation is a byproduct of buyer–seller relationships [12], and the current study aims to identify whether the level of environmentally friendly reputation plays a key role in operationalizing price fairness when implementing RM practices.

## 2. Literature Review

### 2.1. Revenue Management

Generally, researchers suggest that RM consists of two strategic levers: pricing and duration control [13]. While earlier RM strategy in the hospitality industry relied mostly on duration control, pricing strategies have become critical in deploying RM [3]. Technically, hotels consider various factors to price their room rates, but if customers perceive the hotel’s pricing policy as unfair, it leads to decreased customer satisfaction and goodwill, which ultimately loses customers from the business. Past research shows that hotels’ RM practices positively affect customers’ purchase satisfaction when the hotels manipulate their prices fairly [14]. Therefore, it is a hotel’s legitimate role to appease potential customers’ backlash caused by their unfairness perceptions of RM practice.

McMahon-Beattie [9] suggested “revenue management is something that is done to customers rather than something that is done for the customer (p. 44)”. Due to the service firms’ ongoing implementation of RM, it would be one of their core missions to establish mutually valuable and trustworthy relationships with their customers for the purpose of enhancing the two parties’ perceived trust, fairness and justice. However, research remains fragmented on how the outcomes of buyer–seller relationships influence customer perceptions toward RM practice [9]. A firm’s reputation is established from the outcome of sustained relationships between buyer and seller [12] along with an important antecedent of customers’ trust toward the firm [15]. Specifically, the current research examines the impact of service firms’ environmentally friendly reputation on customers’ perceived fairness to bridge gaps in RM literature.

### 2.2. Perceived Price Fairness

When customers believe that the service provider treats them inadequately, they start to judge whether the situation is fair or not [16]. As a psychological factor, customers’ perceived price fairness exerts a significant influence on one’s reactions to prices [17]. Xia et al. [18] define perceived price fairness as one’s judgment and associated emotions of whether the difference (or lack of difference) between sellers’ price and competitors’ prices is reasonable, acceptable, or justifiable. Exploring price fairness issues evolve from the previous study on social exchange [19,20]. Equity theory and distributive justice emphasize the importance of equality of outcomes between two parties in an exchange [7,18,19]. In other words, fairness perceptions are activated when a customer compares an outcome with a comparative other’s outcome [19,21]. This theory further states that disadvantageous inequality is expected to result in anger, while advantageous inequality would lead to feelings of guilt [19,21]. Disadvantageous inequality, nevertheless, is believed to produce stronger negative reactions to the exchange situation than advantageous inequality [19].

Kahneman et al. [17] proposed “the principle of dual entitlement” that largely inspired previous relevant research to further conceptualize individuals’ perceived fairness issues. The principle of dual entitlement indicates that an increase in price is perceived to be fair if it is due to a cost increase but perceived as unfair if it is increased without any increase in cost. This theory further argues that customers are reluctant to pay a price that is perceived as unfair [5,17]. Extending the principle of dual entitlement, Bolton et al. [4] show the impact of three reference points—past prices, competitor prices, and costs—on fairness judgments. Past research also finds that customers who perceive the price as unfair tend to react negatively, such as consumer boycotts, civil action, or lower sales [22]. If hotels increase their room rate, in this sense, during the peak demand period, customers may view this practice as unfair, because a surcharge for room rate is not due to cost increase. The principle of dual entitlement is widely used to explain fairness perceptions in RM practices [3].

If the principle of the dual entitlement holds true, most price discrimination and RM pricing would be seen as unfair, because those pricing practices increase prices without cost increases [6]. However, several ways of increasing price without provoking customer resistance are available [23]. One method is so-called the “rate fences”.

### 2.3. Rate Fences

Assuming that the principle of dual entitlement holds true, the questions of how to increase prices without experiencing customers’ backlash is a critical issue in RM practices. Rate fences are one of the most widely used techniques in RM practice, allowing customers to segment themselves into appropriate rate categories based on their willingness to pay, risk preferences, and behavior [6,24,25,26]. Properly designed rate fences can help service providers effectively target customers with lower prices who are willing to accept certain restrictions on their purchase [6]. In the airline industry, for example, types of fences include requirements for advance purchase and change restrictions, refund penalties, and time of usage restrictions [6,27].

Xia et al. [18] argued that differentially perceived price can induce the perception of advantaged inequality (i.e., the customer who pays less than the reference price or others) or disadvantaged inequality (i.e., the customer who pays more). Adopted from the study of Xia et al. [18], Wirtz and Kimes [3] operationalize rate fences lend into two perspectives: one from the customer paying the higher price that is prevented by an effective rate fence (fence-disadvantaged) and the other from the customer who is able to take advantage of a lower price through the same fencing (fence-advantaged). They further show that customers perceive a fence-advantaged condition much fairer than a fence-disadvantaged condition. Rather than focus on the main effect of rate fence, we will later discuss how such an effect is moderated by the level of a service firm’s environmentally friendly reputation.

### 2.4. Framing Effect

Prospect theory, one of the most important theoretical frameworks in the field of behavioral economics, is introduced to explain the framing effect. This theory explains that individuals are more strongly reactive to losses than to gains [28,29]. People, therefore, “tend to opt for a sure alternative perceived as a gain rather than for a risky alternative of equal expected value, while the converse will hold true for perceived losses” [29]. Based on prospect theory, they further show systematic reversals of preference when the identical problem is given in different forms [30]. Across ten experiments, they show that people are likely to be risk-seeking when encountered with negatively framed situations but to be risk-averse when confronted with positively framed situations. In sum, the framing effect suggests that people evaluate the situations which are framed as positive more favorable than those framed as negative, even if the situations are consequentially identical [30].

Kims and Wirtz [6] apply the framing effect to categorize rate fences into two frames: a discount and a surcharge. In RM practice, they find that customers are more favorable toward a rate framed as a discount than a rate presented as a surcharge [6]. The current study explores the impact of framing on the perceived fairness of rate fence in the service industry. As with the fencing condition, the current study proposes whether this relationship is moderated by environmentally friendly reputation.

### 2.5. Environmentally Friendly Reputation as a Moderator

It is important to understand customer psychology in pricing decisions in order to minimize customers’ perceived unfairness to price differences [31]. Wirtz and Kims [3] confirm that familiarity moderates the effects of framing and fencing conditions on customers’ perceived fairness. Framing as a discount or a surcharge under the conditions of fence advantaged or fence disadvantaged has a significant impact on perceived fairness only for customers who are unfamiliar with the respective RM practice. On the other hand, customers who are familiar with RM practice are not influenced by the framing and fencing conditions [3]. The study of Choi and Mattila [32] also finds that customers’ provision of RM information during their reservation process enhances the perceived fairness of RM practices. Simply giving the information that hotel rates vary, however, is not enough to improve perceived fairness [33]. Alternatively, when customers are provided with information on factors that influence rates (e.g., day of week, length of stay, how far in advance the booking was made), their perception of fairness is likely to improve [32,33].

However, there has been little research on whether a service firm’s environmentally friendly reputation could affect the perception of fairness in revenue management pricing. A critical implication of the current framework is that understanding perceived price fairness in revenue management practices can be increased by the environmentally friendly reputation of service providers. Reputation is based on the service provider’s past actions and has a positive effect on customer satisfaction and loyalty behavior [10,12]. Segarra-Ona et al. argue that a firm’s environmental responsibility is a critical component in business strategy, and it generates both tangible and intangible benefits [34]. In addition, previous research suggests that customers are believed to infer current motives and predict future actions of the service provider with their understanding of the service provider’s past behaviors, as indicated by the service provider’s reputation [11,12]. A significant aspect of reputation is that a good reputation of a service provider is believed to have goodwill value, and a good reputation earns more customer leniency in response to the service provider’s action [11]. A poor reputation, on the other hand, leads to customer distrust and increases the possibility that price increases or differential pricing will encounter complaints from customers in terms of unfairness [35]. A good reputation of the service provider, therefore, may make an equal or advantaged unequal price situation be perceived fairer and decrease customers’ price unfairness perceptions when a disadvantaged price inequality occurs [18].

**Figure 1:**
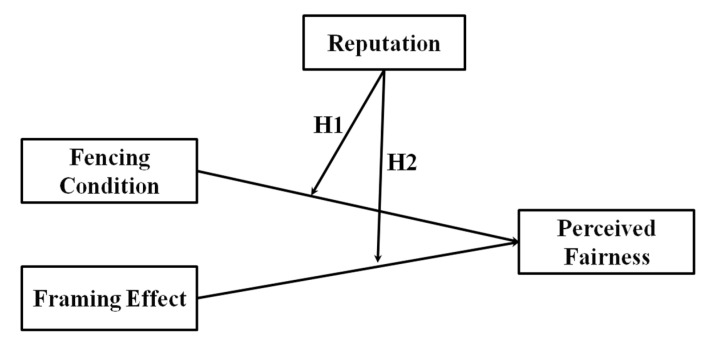
Proposed research model. Notes 1: H1 = Hypothesis 1; Notes 2: H2 = Hypothesis 2.

Applying the notion of reputation to the context of fencing and framing, we expect that a service firm’s environmentally friendly reputation will influence fairness perception in RM practice (see Figure 1 for the research model). Specifically, we hypothesize the reputation-fencing condition (hypothesis 1) and reputation-framing interaction effects (hypothesis 2), respectively. Since a good reputation leads more customer leniency toward a service firm’s action, even in disadvantaged situations [18], we expect no difference in perceived fairness for fencing conditions (fence-advantaged vs. fence-disadvantaged) and framing (discount vs. surcharge), respectively. Conversely, a service firm with a poor reputation will not receive the same leniency, implying that customers will perceive the firm as less fair when they confront with the fence-disadvantaged condition (vs. fence-advantaged) and with the surcharge situation (vs. discount situation), respectively.

**Hypothesis** **1** **(H1).** *When the service firm has a poor environmentally friendly reputation, a fence-advantaged condition will be perceived as fairer than a fence-disadvantaged condition. No such differences in perceived fairness will be expected when the service firm has a good environmentally friendly reputation.*

**Hypothesis** **2** **(H2).** *When the service firm has a poor environmentally friendly reputation, a rate framed as a discount will be perceived as fairer than a rate framed as a surcharge. No such differences in perceived fairness will be expected when the service firm has a good environmentally friendly reputation.*

### 2.6. Proposed Research Model

According to the theoretical background mentioned above, this paper presents a research model as follows:

## 3. Method

### 3.1. Research Design

The current study employed a 2 (framing: surcharge vs. discount) × 2 (fencing: fence-advantaged vs. fence-disadvantaged) × 2 (environmentally friendly reputation: good vs. poor) factorial between-subject experimental design. Adopted from previous research in RM [3,6,36], a scenario-based survey was used for the current research. Eight scenarios were developed to determine the roles of a service firm’s environmentally friendly reputation in RM practice. In addition, the current study employed a self-administered intercept survey with a convenience sampling approach. A total of 268 participants were recruited in the northeast area of U.S. (the states of Massachusetts and New Jersey). Participants were randomly assigned to one of the eight conditions. The target population of the current study was individuals who had booked and stayed hotels in the past. All subjects gave their informed consent for inclusion before they participated in the study. The study was conducted in accordance with the Declaration of Helsinki, and the protocol was approved by the Ethics Committee of University of Massachusetts (3964794).

Participants were first asked to read through the environmentally friendly reputation scenario that presented Hotel ABC (a hypothetical service firm) as having either a good or a poor reputation (see Table 1 for example). The reputation scenarios were directly adopted from the study of [12], a highly cited article in the area of consumer research. After reading one of the reputation scenarios, participants were asked to read one of the four RM scenarios with framing (surcharge vs. discount) and the fencing condition (a customer is in the low-price situation vs. in the high-price situation). Specifically, framing was manipulated at two levels: discount or surcharge. For the discount condition, the room rate reserved 1 month in advance of staying was presented as USD 30 less than the room rate reserved 14 days in advance of staying. For the surcharge condition, the room rate reserved 14 days in advance of staying was presented as USD 30 higher than the room rate reserved 1 month in advance of staying.

The fencing condition was also manipulated at two levels: fence-advantaged and fence-disadvantaged. For the fence-advantaged condition, the customer had paid a lower room rate (i.e., a customer made the reservation 1 month in advance of his or her staying). For the fence-disadvantaged condition, on the other hand, the customer had paid a higher room rate (i.e., a customer made the reservation 14 days in advance of his or her staying). Both framing and fencing scenarios were adopted from the study of Wirtz and Kimes [3].

### 3.2. Dependent Measures and a Covariate

As a dependent variable, perceived price fairness was measured with three-items rated on a 7-point scale anchored by “unfair–fair”, “not at all just–just”, and “unreasonable–reasonable” (adopted from Bolton et al. [4]). Wirz and Kimes [3] argue that customers’ familiarity with RM practices moderates the effects of fencing and framing on perceived fairness. Considering its influence on perceived fairness, we included Wirtz and Kimes [3]’s familiarity scale as a covariate. The proposed hypotheses were tested using analysis of covariance (ANCOVA). While environmentally friendly reputation, price frame, and fencing conditions served as independent variables, familiarity and perceived fairness served as a covariate and a dependent variable, respectively.

## 4. Data Analysis and Results

### 4.1. Descriptive Statistics of Sample

Of a total of 302 respondents established contact, and 268 responses were returned and used for the preliminary data analysis, resulting in a response rate of 88%. The demographic characteristics of the sample in this study are as follows. Of the respondents, 57.1% were female, a majority of respondents (67%) aged between 18 and 25, and 35% had a college degree. The respondents’ average length of staying in hotels last year was 3.4 days. However, 41.8% of respondents stayed more than 7 days, followed by 20.9% between 1 and 2 days and 15.7% between 3 and 4 days.

### 4.2. Manipulation Checks and Reliabilities of Measurement Item

With 30 participants, a pretest was conducted to check whether environmentally friendly reputation scenarios were successfully manipulated; they were randomly assigned to read one of two scenarios of Hotel ABC. Results indicated a significant difference between good and poor reputation scenarios on perceived reputation (F = 4.46, *p* < 0.05). Specifically, the hotel presenting a good reputation resulted in higher evaluations of a hotel’s environmentally friendly reputation (mean = 6.13, where 7 indicates a good reputation) than the hotel presenting a poor environmentally friendly reputation (mean = 1.26). The results, therefore, indicate a successful manipulation of environmentally friendly reputation.

The reliability test was used to test the internal consistency of the measurement items. Two key variables, perceived fairness (three items) and familiarity (two items), were tested, resulting in the Cronbach’s alpha values of 0.94 and 0.85, respectively, which indicate good internal measurement consistency.

### 4.3. Testing Hypothesis

In order to check whether any of participants’ demographic characteristics have effects as covariates, perceived fairness was first analyzed with a 2 × 2 × 2 (environmentally friendly reputation × framing × fencing condition) ANCOVA including gender, age, education level, frequency of staying in a hotel, and the method of making reservations, respectively. Results indicated that none of these factors had any effects as covariates. Thus, they were dropped from further analysis.

The proposed hypotheses were tested using a three-way analysis of covariance (ANCOVA). Table 2 is the mean scores across experimental conditions, and Table 3 shows the ANCOVA table with the covariate, familiarity, included. Considering the significance values, it is clear that the covariate, familiarity of RM practice, significantly predicts perceived fairness. Therefore, customers’ familiarity of RM practices is significantly related to perceived fairness (F = 30.59, *p* < 0.001, η^2^ = 0.106), as shown in the study of [3]. When the effect of familiarity on RM practices is controlled, three main effects of environmentally friendly reputation, fencing condition, and price framing on perceived fairness were statistically significant, F = 31.18, 4.95, and 37.37, respectively (*p* < 0.05). Along with the mean score of perceived fairness, it can be concluded that framing RM pricing as discounts rather than surcharges significantly enhance customers’ perceived fairness. In addition to framing, the fencing condition was also shown to enhance perceived fairness when placing respondents into the fence-advantaged condition than the fence-disadvantaged condition.

### 4.4. Environmentally Friendly Reputation and Fencing Condition Interactions (Hypothesis 1)

**Figure 2:**
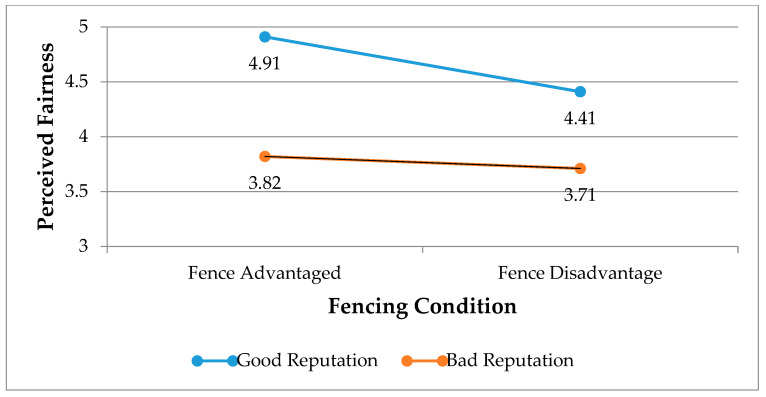
Interaction effects between reputation and fencing condition on perceived fairness.

As shown in Table 3, there were significant main effects of environmentally friendly reputation (F = 31.18, *p* < 0.001, η^2^ = 0.107) and the fencing condition (F = 4.95, *p* < 0.05, η^2^ = 0.019) on perceived price fairness. A hotel with a good reputation, compared to its counterpart, significantly improves customers’ perceived fairness in RM practices. However, the interaction effect between the reputation and the fencing condition was not statistically significant (F = 0.48, *p* > 0.05, η^2^ = 0.002). Specifically, a simple effects test (see Figure 2) showed that fencing did not significantly influence perceived fairness when the hotel had a good reputation ($\bar{x}$ (fence-advantaged/good reputation)) = 4.91 vs. ($\bar{x}$ (fence-disadvantaged/good reputation)) = 4.41, F = 3.47, *p* > 0.05) as well as when the hotel had a poor reputation ($\bar{x}$ (fence-advantaged/poor reputation)) = 3.82 vs. ($\bar{x}$ (fence-disadvantaged/poor reputation)) = 3.71, F = 0.02, *p* > 0.05). Taken together, these results fail to support H1.

A possible explanation for these results is that individuals tend to have predetermined views of social norms which in turn affect fairness perceptions of firms’ practices [36,37,38]. Although a firm’s certain practice is initially perceived as unfair, it slowly evolves into social norms over time, which in turn is less likely to be perceived as unfair [17]. Since RM practices have been exercised for more than 30 years, there might be a possibility that the practice of rate fences is perceived as less unfair over time. Although the main effect of fencing condition is significant, only 1.9% (i.e., η^2^ = 0.019) of the total variance in a dependent variable (i.e., perceived fairness) is accounted for by fencing conditions, indicating a weak effect on perceived fairness [39]. In the ANCOVA model, effect size estimates the magnitude of effect on an outcome variable and should be taken into account, in addition to the *p*-value [40,41,42]. Thus, it might be possible that customers see a practice of different rate fences (i.e., fence-advantage vs. fence-disadvantaged conditions) as a social norm, and therefore they are not influenced by a hotel’s environmentally friendly reputation when judging price fairness of rate fences.

### 4.5. Environmentally Friendly Reputation and Framing Interaction (Hypothesis 2)

**Figure 3:**
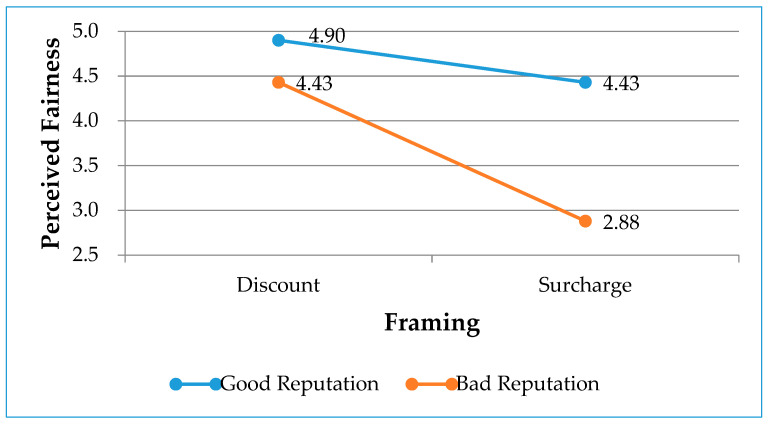
Interaction effects between reputation and framing on perceived fairness.

As shown in Table 3, there were significant main effects of environmentally friendly reputation (F = 31.18, *p* < 0.01, η^2^ = 0.107) and framing (F = 37.37, *p* < 0.01, η^2^ = 0.126) along with a significant interaction effect between environmentally friendly reputation and framing (F = 12.05, *p* < 0.01, η^2^ = 0.044). Follow-up simple effect analyses revealed that the main effects were qualified by the interaction. In particular, framing did not significantly affect perceived fairness when the hotel had a good reputation ($\bar{x}$ (discount/good reputation)) = 4.90 vs. ($\bar{x}$ (surcharge/good reputation)) = 4.43, F = 4.19, *p* > 0.05). When the hotel had a poor environmentally friendly reputation, however, price framed as a discount rather than a surcharge substantially increased the perceived fairness ($\bar{x}$ ((discount/poor reputation)) = 4.43 vs. ($\bar{x}$ (surcharge/poor reputation)) = 2.88, F = 39.87, *p* < 0.001), which supports Hypothesis 2. The mean scores plotted in Figure 3 confirm this, as operationalized in Hypothesis 2.

## 5. Discussion and Implication

The impact of firms’ environmentally friendly actions has been studied across various disciplines. For example, Chan and Ng (2020) examined how message characteristics and user factors influence environmentally friendly message design [43]. In addition, Hwang et al. (2020) adopted the concept of a green image in the context of eco-friendly edible insect restaurants [44]. Despite both the theoretical and managerial importance of the topic, scant research has been conducted in the context of revenue management. Drawing upon psychological and economic theories, therefore, the current study examines the effects of a service firm’s environmentally friendly reputation in the RM context. First, we explore whether framing RM pricing as discounts rather than surcharges would significantly enhance customers’ perceived fairness. The results of this study indicate that the framing of RM practice as a discount rather than a surcharge makes customers seem fairer-treated and would, therefore, be less likely to result in negative customer perceptions. This finding supports the framing effect [30] in that individuals evaluate the situations which are framed as positive more favorable than those framed as negative, even if the situations are consequentially identical.

In addition to framing, the current study confirms that the fencing condition is an additional variable that determines perceived fairness in RM practice. Participants who were placed in a fence-advantaged condition perceived RM practice fairer than a fence-disadvantaged condition. However, the effect size of a fencing condition, compared to that of a framing condition, is substantially small, indicating a weak effect on perceived fairness. In addition, the result shows that there is no interaction effect between fencing conditions and environmentally friendly reputation, indicating that such a reputation does not moderate the effect of fencing conditions on perceived fairness. Therefore, the study cannot confirm Hypothesis 1.

On the other hand, Hypothesis 2 is supported in this study. The analysis finds that there is an interaction effect of environmentally friendly reputation and framing. Specifically, the reputation moderates the effect of framing on perceived fairness. When the service firm has a poor reputation, framing as a discount rather than framing as a surcharge considerably increases customers’ perceived fairness. When the service firm has a good reputation, however, there is no significant difference between priced framed as a discount and a surcharge. The finding suggests an important way in which a good environmentally friendly reputation can be beneficial to service firms when they implement RM practice. A possible explanation for observed environmentally friendly reputation and framing interaction is the service firm’s environmentally friendly reputation makes customers believe that a good reputation has “goodwill” value even if the service firm’s price is framed as a surcharge [11]. A good environmentally friendly reputation, in other words, is expected to gain greater leniency from customers and soften customers’ reactions to a firm’s action [12]. Specifically, the goodwill value of a good environmentally friendly reputation is such that the customers infer the motive of the service firm for implementing price differentiation strategy positively. In contrast, customers believe that a poor reputation does not have “goodwill”, thus they infer that the service firm has negative motivation for implementing RM practice [15]. The findings of this study imply that service firms strive to build good and positive environmentally friendly reputations in customers’ minds for the purpose of deriving their favorable attitude towards hotels’ nontraditional operational practices such as RM practices.

As with any study using the experimental design, this study has some limitations. Although the experiments allow the close and careful examination of the factors of interest, they also involve some artificiality [12]. For instance, the hotel’s environmentally friendly reputation scenario was provided to participants in a short, written format, which is somewhat unrealistic. In addition, the scenario of reputation may have been more dynamic, instead of being inert. In the case of the poor reputation scenario, all descriptions were fairly negative, whereas in a real case, there is a combination of negative, neutral, and positive aspects about a hotel [12].

The current study employs an environmentally friendly reputation as a moderator in the context of perceived fairness of RM practice. Additional research should explore the psychological mechanism (i.e., mediating variables) that links reputation to perceived fairness. For example, it could be emotional distress, trustworthiness [45] or feelings of betrayal [46] which mediate the relationship between reputation and perceived fairness. In addition, future research should explore other potential moderating variables such as individuals’ sense of power. Jin et al. [47] argue that high-power consumers show greater perceptions of price unfairness when they receive a disadvantaged price relative to other consumers. This might be evidence that high-power consumers are more sensitive to fencing conditions than low-power consumers.

## 6. Conclusions

In revenue management practice, the issue of perceived fairness is a serious concern since RM applies different prices for basically the same service. When various prices are charged for fundamentally the same service, customers are likely to compare their paid prices with other customers’ paid prices. Thus, it is important to understand what factors influence customers’ perceptions of fairness in revenue management pricing. To that end, the current study examines how a firm’s environmentally friendly reputation alter customers’ fairness perceptions. The findings of this study help firms to understand how customers perceive fairness in revenue management practice.

## Figures and Tables

**Table 1:** Example of environmentally friendly reputation conditions.

Good Environmentally Friendly Reputation
Hotel ABC is a large hotel chain that has been established for many years and is highly regarded worldwide. This hotel appears to treat both its customers and its employees with respect. Employees feel that the hotel tries to be equitable in its interactions and negotiations. Customers state that this hotel seems to have a philosophy of providing good service. Moreover, Hotel ABC participates in various community activities and engages in sustainable practices that conserve natural resources and reduce environmental impact.

**Table 2:** The mean score of fairness perceptions.

	Good Reputation Mean (SD)		Poor Reputation Mean (SD)	
Price Frame	Discount	Surcharge	Discount	Surcharge
Fence-advantaged	5.13 (1.16)	4.66 (1.33)	4.81 (1.17)	2.93 (1.54)
Fence-disadvantaged	4.61 (1.24)	4.17 (1.34)	4.18 (1.51)	2.82 (1.44)

Note. A 7-point scale from 1 = ‘very unfair’ to 7 = ‘very fair’.

**Table 3:** Analysis of covariance (ANCOVA) results on perceived fairness.

Source of Variation	df	Sum of Square	Mean Square	F	*p*	Eta Square
Familiarity	1	50.412	50.42	30.59	<0.001	0.106
Reputation	1	51.38	51.38	31.18	<0.001	0.107
Fencing	1	8.15	8.15	4.95	<0.05	0.019
Framing	1	61.58	61.58	37.37	<0.001	0.126
Reputation × Fencing	1	0.78	0.78	0.48	ns	0.002
Reputation × Framing	1	19.85	19.85	12.05	<0.01	0.044
Fencing × Framing	1	0.021	0.021	0.013	ns	0.000
Reputation × Fencing × Framing	1	0.045	0.045	0.028	ns	0.000
Error	259	426.77	1.65			
Total	268	5355.56				
Corrected Total	267	641.47				

Notes 1: df = degree of freedom; Notes 2: ns = not significant.
